# Myelodysplastic Syndromes in the Postgenomic Era and Future Perspectives for Precision Medicine

**DOI:** 10.3390/cancers13133296

**Published:** 2021-06-30

**Authors:** Ioannis Chanias, Kristina Stojkov, Gregor Th. Stehle, Michael Daskalakis, Helena Simeunovic, Linet Muthoni Njue, Annatina S. Schnegg-Kaufmann, Naomi A. Porret, Ramanjaneyulu Allam, Tata Nageswara Rao, Rudolf Benz, Axel Ruefer, Adrian Schmidt, Marcel Adler, Alicia Rovo, Stefan Balabanov, Georg Stuessi, Ulrike Bacher, Nicolas Bonadies

**Affiliations:** 1 Department of Hematology and Central Hematology Laboratory, Inselspital, Bern University Hospital, University of Bern, 3010 Bern, Switzerland; ioannis.chanias@insel.ch (I.C.); kristina.stojkov@insel.ch (K.S.); michael.daskalakis@insel.ch (M.D.); helena.simeunovic@insel.ch (H.S.); LinetMuthoni.Njue@insel.ch (L.M.N.); Annatina.Schnegg@insel.ch (A.S.S.-K.); NaomiAzur.Porret@insel.ch (N.A.P.); allam.ramanjaneyulu@dbmr.unibe.ch (R.A.); tata.nageswararao@dbmr.unibe.ch (T.N.R.); Alicia.Rovo@insel.ch (A.R.); veraulrike.bacher@insel.ch (U.B.); 2 Department for BioMedical Research (DBMR), University of Bern, 3010 Bern, Switzerland; 3 Clinic of Hematology, University Hospital Basel, 4031 Basel, Switzerland; GregorThomas.Stehle@usb.ch; 4 Department of Hematology and Oncology, Hospital Thurgau AG, 8596 Muensterlingen, Switzerland; rudolf.benz@stgag.ch; 5 Department of Hematology and Central Hematology Laboratory, Cantonal Hospital Lucerne, 6004 Lucerne, Switzerland; axel.ruefer@luks.ch; 6 Department of Internal Medicine, Clinic of Medical Oncology and Hematology, City Hospital Waid and Triemli, 8063 Zurich, Switzerland; Adrian.Schmidt@triemli.zuerich.ch; 7 Center for Medical Oncology and Hematology, Hospital Thun, 3600 Thun, Switzerland; marcel.adler@spitalstsag.ch; 8 Department of Medical Oncology and Hematology, University Hospital Zurich, University of Zurich, 8091 Zurich, Switzerland; stefan.balabanov@usz.ch; 9 Clinic of Hematology, Oncology Institute of Southern Switzerland, 6500 Bellinzona, Switzerland; Georg.Stuessi@eoc.ch

**Keywords:** myelodysplastic syndromes, postgenomic era, precision medicine, targeted therapies, future perspectives

## Abstract

**Simple Summary:**

With demographic ageing, improved cancer survivorship and increased diagnostic sensitivity, incident cases of patients with Myelodysplastic Syndromes (MDS) are continuously rising, leading to a relevant impact on health care resources. Disease heterogeneity and various comorbidities are challenges for the management of the generally elderly patients. Therefore, experienced physicians and multidisciplinary teams should be involved in the establishment of the correct diagnosis, risk-assessment and personalized treatment plan. Next-generation sequencing allows for early detection of clonal hematopoiesis and monitoring of clonal evolution, but also poses new challenges for its appropriate use. At present, allogeneic hematopoietic stem cell transplantation remains the only curative treatment option for a minority of fit MDS patients. All others receive palliative treatment and will eventually progress, having an unmet need for novel therapies. Targeting compounds are in prospect for precision medicine, however, abrogation of clonal evolution to acute myeloid leukemia remains actually out of reach.

**Abstract:**

Myelodysplastic syndromes (MDS) represent a heterogeneous group of clonal disorders caused by sequential accumulation of somatic driver mutations in hematopoietic stem and progenitor cells (HSPCs). MDS is characterized by ineffective hematopoiesis with cytopenia, dysplasia, inflammation, and a variable risk of transformation into secondary acute myeloid leukemia. The advent of next-generation sequencing has revolutionized our understanding of the genetic basis of the disease. Nevertheless, the biology of clonal evolution remains poorly understood, and the stochastic genetic drift with sequential accumulation of genetic hits in HSPCs is individual, highly dynamic and hardly predictable. These continuously moving genetic targets pose substantial challenges for the implementation of precision medicine, which aims to maximize efficacy with minimal toxicity of treatments. In the current postgenomic era, allogeneic hematopoietic stem cell transplantation remains the only curative option for younger and fit MDS patients. For all unfit patients, regeneration of HSPCs stays out of reach and all available therapies remain palliative, which will eventually lead to refractoriness and progression. In this review, we summarize the recent advances in our understanding of MDS pathophysiology and its impact on diagnosis, risk-assessment and disease monitoring. Moreover, we present ongoing clinical trials with targeting compounds and highlight future perspectives for precision medicine.

## 1. Epidemiology

MDS is a heterogeneous group of clonal conditions arising from somatic mutations in hematopoietic stem and progenitor cells (HSPCs), mainly affecting elderly individuals [1]. Ineffective hematopoiesis in MDS is characterized by a vicious circle of maturation defects (dysplasia), inflammation in the bone marrow (BM) microenvironment and cytopenia in peripheral blood (PB), which is accompanied by variable risk of progressing towards secondary Acute Myeloid Leukemia (sAML) [2]. The median age at presentation is above 70 years, with an age-standardized incidence-rate of 3–5 cases per 100,000 patient-years and a prevalence of 20 patients per 100,000 individuals [3]. The age-specific incidence-rate increases progressively with age, with 50 cases per 100,000 patient-years in individuals 75 years [3,4]. Males are predominantly affected, with the exception of MDS with isolated del(5q), which is more frequent in females. Therapy-related MDS is estimated to represent 10% of all MDS cases, though precise incidence rates cannot be determined from current epidemiological data [5,6]. Although generally a disease of the elderly, MDS can occur at any age. The presence of genetic predisposition syndromes should be thoroughly investigated in childhood or younger adults (40 years). In such cases, multiple organs can be affected, and these individuals carry a risk for increased toxicity to chemotherapy and development of other cancers [7,8].

## 2. Pathophysiology

### 2.1. Recurrent Somatic Leukemia-Associated Driver Mutations and Clonal Hematopoiesis

**Figure 1:**
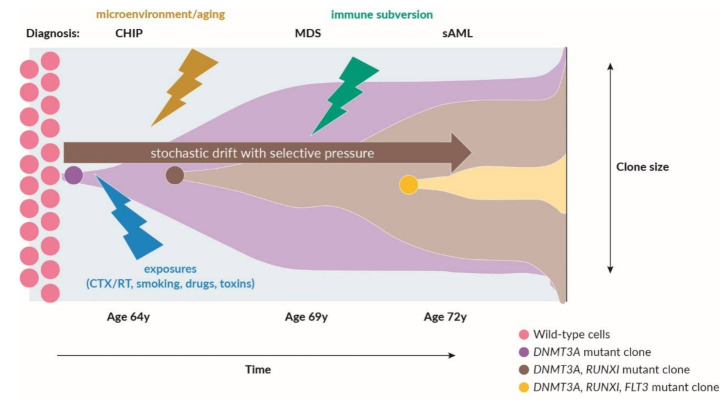
Factors involved in clonal evolution (adapted from [16]). CHIP: clonal hematopoiesis of indeterminate potential; MDS: myelodysplastic syndromes; sAML: secondary AML; CTX: chemotherapy; RT: radiotherapy. Adapted from [23].

Next-generation sequencing (NGS) has revolutionized our understanding of the genetic landscape in MDS. Nonetheless, the precise molecular mechanisms involved in clonal dominance and evolution remain unclear [9]. NGS allowed the identification of recurrent somatic leukemia-associated driver mutations (SLADMs) in genes that are classified into RNA-splicing factors, epigenetic regulators, transcription factors, cell-cycle regulators, cohesin complex factors, as well as cell-signaling molecules (Table 1) [10,11,12,13,14,15]. Evolution from clonal hematopoiesis to MDS and sAML is caused by sequential accumulation of random genetic hits in HSPCs with many mechanisms involved. Genetic drift originates from cell-intrinsic growth advantage, reduced cell-death and selective pressure imposed by a variety of cell-extrinsic factors. These can include radiation, chemotherapy, cytotoxic drugs and many other toxins (i.e., benzol exposition) [16]. Further genetic drift can be promoted by coexisting intrinsic DNA repair defects, loss of immunogenic tumor surveillance, remodeling of the BM microenvironment and other niche-factors associated with ageing [17] (Figure 1). Age-related clonal hematopoiesis (ARCH) and clonal hematopoiesis of indeterminate potential (CHIP) are defined by the presence of SLADMs at a variant allele frequency (VAF) ≥2% in individuals without cytopenia or other signs of hematologic disease. CHIP is a relevant phenomenon observed in the ageing population, affecting 20–40% of individuals 80 years, and associated with an increased risk for transformation to overt hematological malignancies [18,19,20,21,22] (Table 2). The term *Clonal Cytopenia of Unknown Significance* (CCUS) refers to individuals with cytopenia and clonal hematopoiesis that do not fulfill the formal diagnostic criteria for MDS [19]. As clonal hematopoiesis is a frequent condition, the borderlines between facultative clonal pre-cancerous conditions, non-neoplastic aplastic anemia and overt myeloid malignancies are increasingly blurred. The types of genes affected by somatic mutations, numbers of mutations and size of the mutated clone estimated by the VAF may help to distinguish these entities (Table 3).

### 2.2. The Role of Adaptive and Innate Immunity in MDS

Clonal hematopoiesis is associated with increased cardiovascular morbidity, including premature atherosclerosis and pathological cardiac remodeling as well as other chronic inflammatory or degenerative disorders of ageing [27]. Cardiovascular, inflammatory and autoimmune conditions are more frequent in MDS patients compared to the normal population [28,29]. However, it remains unclear whether these are off-target or disease-driving effects. The switch from an activated to exhausted immunological tumor surveillance, referred to as immune subversion, is characteristic for neoplastic conditions and promotes further clonal expansion [30]. Although this immunological phenomenon needs further understanding, it opens the field for early therapeutic interventions aiming to revert immune subversion and reduce progression to higher-risk MDS or sAML.

Recent translational studies suggest that dysregulation of the innate immunity and an associated hyper-inflammatory state contribute to the pathogenesis of lower-risk MDS [31]. Increased levels of cytokines, chemokines and growth factors have been observed in PB and BM of MDS patients and are associated with adverse clinical outcomes [32,33,34]. Furthermore, larger-scale epidemiologic studies showed that patients with autoimmune disorders have an increased risk of developing MDS [35,36]. Innate immune receptors, such as Toll-like receptors (TLRs), participate in the pathogenesis of several non-infectious, inflammatory and autoimmune disorders [37]. Several studies suggest that a number of TLRs, as well as other signal transducers in this pathway, are overexpressed in a high proportion of MDS patients (40–80%) [31,38]. Although, the precise role of TLR-mediated signaling in MDS remains to be fully elucidated, in vitro and in vivo studies suggest that this pathway is involved in the loss of progenitor cell function leading to impaired differentiation of HPSCs. In line with this, a recent study highlighted the crucial role for NLR family pyrin domain containing 3 (NLRP3) inflammasome in lower-risk MDS pathology and propagation of clonal HSPCs [39]. Inflammasomes are cytosolic innate immune receptors that upon activation form caspase-1-activating multiprotein complexes. These activate interleukin-1 cytokine members (IL-1β and IL-18) and initiate Gasdermin-D mediated pyroptosis, an inflammatory form of cell death [40,41]. Therefore, pyroptosis and immune subversion play mechanistically an important role in lower- and higher-risk MDS, respectively, and are currently under investigation as potential treatment targets.

## 3. General Aspects of MDS Patient Management

The heterogeneity of MDS and the multimorbidity represent major challenges. The disease course may vary from chronic asymptomatic or minimal symptomatic cytopenia to rapid progression towards sAML. Therefore, correct diagnosis, disease- and patient-based risk stratification are essential for an appropriate treatment plan. Experienced physicians, acting within interdisciplinary diagnostic and therapy review boards, should preferentially be involved. Lower-risk MDS patients have a median survival of 3 to 8 years and mostly succumb to non-leukemic causes of death. These include mainly cardiovascular events, infections and other relevant comorbidities, being aggravated by cytopenia and inflammation. Thus, treatment in lower-risk MDS should improve symptomatic cytopenia and optimize comorbidities aiming to improve quality of life (QoL) and delay progression [42,43]. Higher-risk MDS patients have a median survival of 1 to 3 years and die predominantly of complications related to sAML progression. The treatment aim in these patients is the reduction of progression and improvement of overall survival (OS) with minimal treatment-related toxicities [44,45].

## 4. Diagnostic Approach and Risk-Stratification

In patients with suspected MDS, previous exposure to genotoxic agents (e.g., cytotoxic chemotherapy, radiation) should be evaluated, which indicates the presence of a therapy related myeloid neoplasm (t-MN). Younger MDS patients (40 years) should be thoroughly screened for germline predispositions, which may be indicated by a family history of malignancies as well as immune or organ dysfunctions in first- and second-degree relatives. The European Leukemia Net recommendation recognizes diagnostic procedures as “mandatory” (evaluation of PB smears and BM aspirate/biopsy with cytogenetic analysis), “recommended” (fluorescence in situ hybridization (FISH) and flow cytometry), and “suggested” in specific circumstances (single-nucleotide-polymorphism (SNP), molecular diagnostics) [46]. Process-based indicators as measurable elements for quality of care are of increasing interest to enable assessment and comparison of the impact of different health care environments on relevant MDS outcomes [47].

### 4.1. WHO Classification and Minimal Diagnostic Criteria for MDS

MDS are diagnosed according to the updated classification of the WHO 2016 (Table 4) [48]. Cytopenia and dysplasia remains the mainstay in the diagnosis of MDS; however, assessment of dysplasia is subjective with inter-individual variability (Table 5) [49]. Some patients with persisting cytopenia for at least 6 months may fail to fulfill these criteria. Therefore, an international working group proposed minimal diagnostic criteria and co-criteria that define conditions with high suspicion for myeloid neoplasm or MDS. These include BM stem cell proliferation, aberrant immunophenotypic characteristics, clonality of myeloid cells as well as abnormal gene-expression profiles (Table 6) [24]. Cases that do not meet the co-criteria of clonality are classified as idiopathic cytopenia or dysplasia of undetermined significance (ICUS/IDUS) [50].

### 4.2. Role of NGS in MDS Diagnosis, Follow-Up and Risk-Stratification

**Figure 2:**
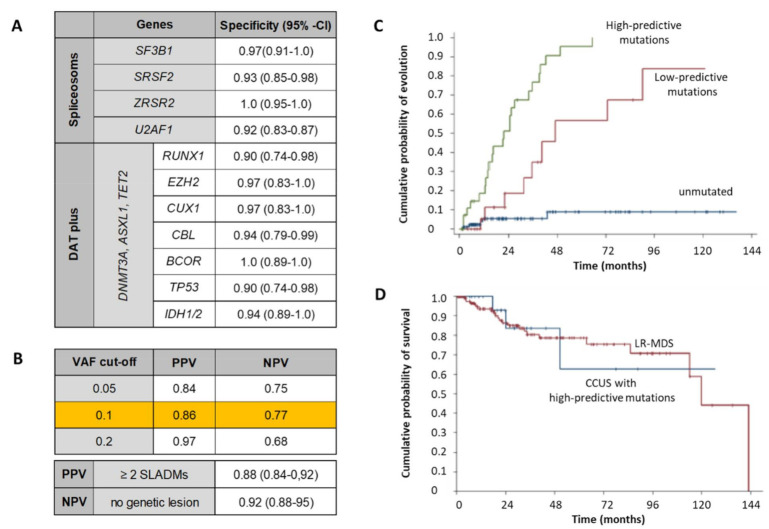
Predictive mutations for myeloid malignancies (adapted from [53]). (**A**) Higher-risk SLADMs (spliceosome genes and DAT plus mutations) with their specificity for development of myeloid malignancy over 5 years. (**B**) Variant-allele frequency (VAF) cut-offs with their corresponding positive and negative predictive values (PPV, NPV) for evolution to myeloid malignancy over 5 years. PPV and NPV at the bottom of the table are represented for a cut-off VAF at 0.1. (**C**) Cumulative probability for evolution to myeloid malignancy for high- (green), low-predictive (red) and unmutated (blue) individuals. (**D**) Cumulative probability of survival for individuals with clonal cytopenia of undetermined significance (CCUS) with high-predictive mutations (blue) and lower-risk MDS (red: LR-MDS).

Targeted NGS panels offer the analysis of hotspot mutations in 40–50 genes at a sensitivity of ~5% VAF [51,52]. This allows the identification of clonal hematopoiesis at early stages of development. At the same time, it becomes increasingly difficult to distinguish age-related changes with a favorable course from conditions that progress more rapidly to overt hematological malignancies. The risk for progression depends on the affected genes (higher-risk as compared to lower-risk mutations), the number of SLADMs and the clonal burden (10% VAF). Patients with unclear cytopenia and higher-risk SLADMs have a similar probability of survival as lower-risk MDS patients (Figure 2) [53]. In the case of stable clonal hematopoiesis, thorough clinical and laboratory observation may be indicated every 3−6 months, with repeated BM assessment at signs of progression (worsening cytopenia, occurrence of cytosis, blasts or precursors in PB) [46]. NGS has revolutionized diagnostics, risk stratification and treatment monitoring in MDS. However, controversies and challenges remain on its rational use in MDS [54,55,56,57]. Genes can also be mutated constitutionally (germline) and may indicate the presence of predisposition syndromes for myeloid malignancies (Table 1). NGS has not only gained importance in identifying the clonal origin of unclear cytopenias, but it also allows us to identify potential therapeutic targets (*SF3B1*, *TP53*, *IDH1/2*). Moreover, it plays an important role in prognosis as well as monitoring after allogeneic hematopoietic stem cell transplantation (allo-HCT) [54,55]. More recently, genomic features of cytogenetics and NGS have been integrated for next-generation disease classification and prognostication based on biological information [58]. Sequential NGS analysis may gain importance for the assessment of clonal composition during treatment and for the genetically inferred selection of targeting compounds to refractory subclones in the near future.

### 4.3. Hypoplastic MDS and Aplastic Anemia

The presence of unexplained cytopenia accompanied by signs of dysplasia in the PB or BM and, at later stages, increase of myeloid blasts, is seminal for the diagnosis of MDS. This is especially challenging in conditions with hypoplastic BM (cellularity 30%), referred to as hypoplastic MDS (hMDS) and occurring in 5–10% of all MDS cases [4]. When cytomorphology is not sufficient to confirm or exclude hMDS, cytogenetics may identify clonality with detectable chromosomal abnormalities in ~50% of cases. NGS increases the sensitivity for the identification of clonality. However, SLADMs can also be found in aplastic anemia (AA), thus making the distinction form hMDS more challenging, adding more complexity in finding the correct diagnosis. An integrated cyto-histologic/genetic score (hg-score) has been recently developed to facilitate distinction between AA and hMDS (Table 7) [53,59]. Other conditions may mimic hMDS or AA and etiologies can be multifactorial in elderly patients, such as transient aggravation of cytopenia during infections or drug-exposure in patients with CHIP. Delay in recovery after these intercurrences suggest conditions that are more advanced, directing further investigations to exclude MDS.

### 4.4. Disease-Based Risk Stratification

Different scoring systems support clinical decision making by estimating the risk for progression to sAML and OS. The International Prognostic Scoring System (IPSS) [60], the revised IPSS (IPSS-R) [61] and the WHO Prognostic Scoring System (WPSS) [62] are the most widely used scoring systems. The WPSS seem to be less important according to a recent consensus for defining relevant indicators [47]. Lower-risk MDS is generally characterized by mild and single-lineage cytopenia, blasts 5% and the presence of a normal karyotype or favorable cytogenetic aberrations. In contrast, more severe and multi-linage cytopenia, transfusion-dependency, excess of blasts as well as poor-risk or complex cytogenetic aberrations characterize higher-risk MDS. Somatic mutations in *TP53*, *EZH2*, *ETV6*, *RUNX1*, *ASXL1*, *SRSF2*, *U2AF1*, *RAS*-pathway and *JAK2* with VAF ≥2% provide independent prognostic information, but are not yet integrated in current scoring systems [13]. However, information about the mutation profile in individual patients can be clinically meaningful and further supports the implementation of targeted NGS analysis, particularly for younger MDS patients.

### 4.5. Patient-Based Risk Stratification

Patient-based risk stratification considers age, comorbidities, performance status and frailty (reduced physical fitness) to estimate their impact on treatment-related mortality. Karnofsky and ECOG performance scores are broadly applied to assess residual functional ability [46]. Using individualized patient-based risk-stratification, MDS patients are classified as fit (good performance status without limiting comorbidities) or unfit (poor performance status and/or multiple comorbidities) for intensive treatment approaches, including standard induction chemotherapy or allo-HCT. The hematopoietic stem cell transplant-specific comorbidity index (HCT-CI) allows the prediction of non-relapse mortality in the allo-HCT setting [63]. The MDS-specific comorbidity index (MDS-CI) is a simplified form of the HCT-CI (cardiac, hepatic, pulmonary, renal disorders and previous solid tumors) and more frequently used in transplant ineligible MDS patients [64]. MDS-specific frailty index adds independent prognostic information to the IPSS-R score [65]. Patient-based risk factors have an independent impact on OS of elderly patients with MDS and should be used together with disease-based risk stratification [47,66].

### 4.6. Patient-Reported Outcomes (PROs)

The Quality of life (QoL) and symptom-burden including pain/discomfort, immobility, anxiety/depression, and most commonly fatigue are inferior in MDS patients compared to age-matched controls [8]. Fatigue by itself is frequent in MDS and has a negative prognostic impact on survival [67]. A variety of QoL assessment tools are available, including the European Organization for Research and Treatment of Cancer Quality of Life Questionnaire (EORTC QLQ-C30), EuroQol 5 Dimension (EQ-5D), the Functional Assessment of Cancer Therapy Anemia Scale (FACT-An) and the MDS-specific Quality of Life in Myelodysplasia Scale (QUALMS) [68,69,70,71]. These instruments integrate functional factors (physical, role, cognitive, emotional, and social), symptoms (fatigue, pain, nausea and vomiting), global health as well as QoL items. Incorporating patient-reported outcomes (PROs) is generally considered relevant for individualized MDS treatment. However, a generally accepted gold standard and the therapeutic impact remains currently unclear [47]. Moreover, many tools seem to be impracticable for the daily routine and simplified screening tools or instruments focusing on self-sufficiency in nutrition or mobility may be valuable alternatives [72].

## 5. Therapeutic Approach

**Figure 3:**
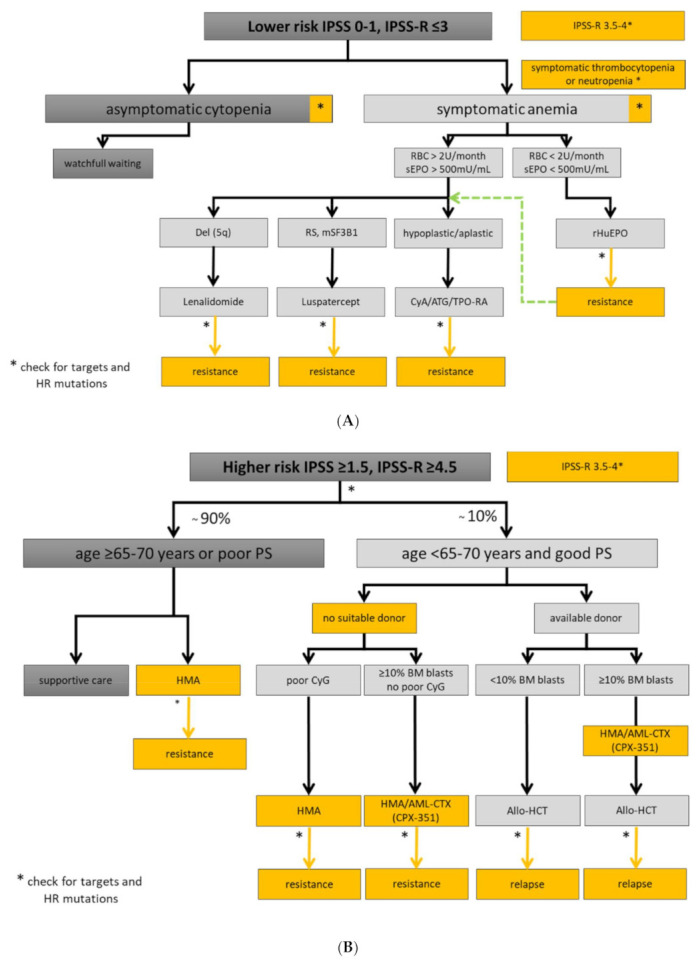
Treatment algorithm for lower- and higher-risk MDS. Yellow boxes highlight areas with an unmet clinical need in in lower (**A**) and higher-risk MDS (**B**). High-risk (HR) mutations comprise: ≥3 SLADMs or single mutations in *TP53*, *RUNX1*, *ASXL1*, *ETV6*, *EZH2*, *SRSF2*, *U2AF1*, *RAS*-pathway and *JAK2* with VAF ≥2% [13,74,75]. Allo-HCT: allogeneic hematopoietic stem cell transplantation; AML-CTX: AML-based chemotherapy; ATG: antithymocyte globulin; BM: bone marrow; CSA: Cyclosporine A; CyG: cytogenetics; ESA: erythropoietin stimulating agent; HMA: hypomethylating agent; HR: high-risk mutations; *mSF3B1*: mutated *SF3B1*; PB: peripheral blood; PS: performance status; RBC: red blood cell concentrate; RS: ring sideroblasts; sEpo: serum erythropoietin; TPO-RA: thrombopoietin receptor agonist. Adapted from [23].

**Figure 4:**
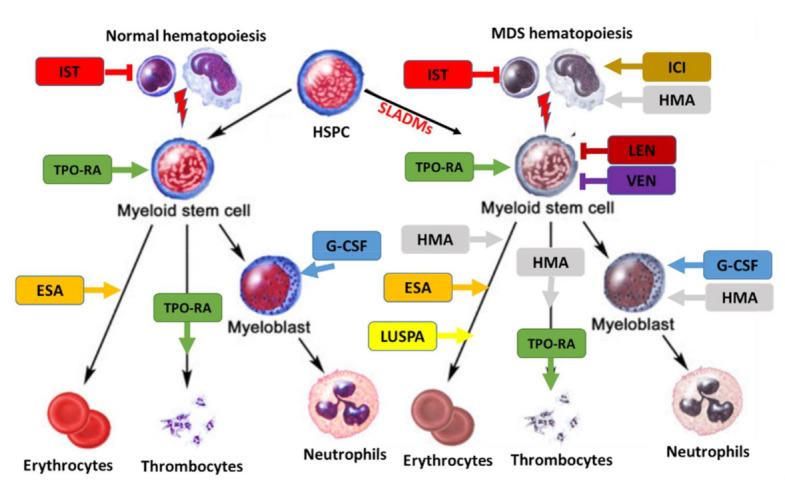
Treatment landscape in MDS. Treatment can affect normal (**left**) and clonal hematopoiesis (**right**). SLADMs: somatic leukemia-associated driver mutations; ESA, erythropoietin stimulating agent; ICI, immune checkpoint inhibitors; G-CSF, granulocyte colony stimulating factor; HMA, hypomethylating agents; IST, immune suppressive treatment (CyA/ATG); LEN, lenalidomide; LUSPA, luspatercept; TPO-RA: thrombopoietin receptor agonists; VEN, venetoclax. Adapted from [23].

Experienced physician and interdisciplinary boards should be involved in the assessment of MDS patient with symptomatic cytopenia or unexplained inflammatory conditions. This is important due to disease complexity, interfering comorbidities, and timely selection of higher-risk MDS patients being eligible for allo-HCT. Fluctuating cytopenias may be the initial manifestation of clonal hematopoiesis. However, patients with uncharacterized systemic autoinflammatory manifestations may also present with SLADMs at high VAFs, as primary manifestation of clonal hematopoiesis or even MDS [41]. New therapeutic options and clinical trials are urgently needed, especially in elderly patients with refractory conditions [73]. Therefore, symptomatic patients should be referred to experienced MDS centers, included in prospective registries/biobanks, and offered participation to clinical trials, whenever possible. The therapeutic approaches for lower- and higher-risk MDS are summarized in Figure 3A,B, respectively, and an overview on the treatment landscape can be found in Figure 4. 

### 5.1. Lower-Risk MDS Patients

#### 5.1.1. Watchful Observation and General Supportive Treatment

Life expectancy of asymptomatic MDS patients 70 years of age with single lineage dysplasia (SLD) or del(5q) does not substantially differ from an age-matched population [62]. Therefore, watchful observation is adequate for most asymptomatic MDS patients. Despite increasing knowledge of high-risk genetic constellations [13,74,75], no prospective clinical trial could demonstrate any benefit for early interventions in these patients, and supportive treatment is still the mainstay of lower-risk MDS patients. Non-disease related factors like nutrition and functionality are often overlooked or not specifically addressed despite their prognostic importance [76]. The same is true for psychological and social distress that may influence compliance and treatment adherence.

#### 5.1.2. Treatment of Anemia

Transfusions of red blood cells (RBC) can improve symptomatic anemia immediately. A hemoglobin threshold 80 g/L is often applied but should be individualized depending on age, comorbidities and symptoms. Repeated transfusions can cause alloimmunization and iron overload. Iron chelation therapy is usually recommended in patients with 20 transfused RBCs, serum ferritin 1000 µg/L or other signs of iron-overload with a life expectancy 1 year or candidates for allo-HCT [46,77,78]. Yet, the indication remains somehow controversial, since a recently performed prospective clinical trial with deferasirox could only show a reduction of event-free survival (cardiovascular events). Due to low accrual, the trial had to be closed prematurely and the power was not sufficient to show a reduction in OS [47,79,80,81,82,83,84]. Erythropoietin stimulating agents (ESA) can improve symptomatic anemia and delay transfusions. Best responses can be achieved in lower-risk MDS patients with an endogenous serum erythropoietin 500 U/L and low transfusion burden (≤4 RBCs over 8 weeks) (Nordic criteria) [46,85,86]. While all erythropoietin agents seem to be similarly efficient, randomized, placebo-controlled trials exist only for epoetin alpha (450 IU/kg every week) and darbepoetin alpha (300–500 µg every 2 to 3 weeks) [87,88]. Responses range around 40–50% with a median duration of 1–2 years and poor prognosis for ESA refractory patients [89]. Addition of granulocyte-colony stimulating factor (G-CSF) to ESA in anemic MDS patients is generally recommended, but remains controversial, as current data is limited for an additional efficacy, if added to full-dose ESA [90]. Moreover, the combination of ESA with lenalidomide (LEN) seems to increase the response-rates in ESA refractory lower-risk MDS patients [91]. Luspatercept (LUSPA) is a first in class erythroid maturating agent (EMA) interfering with aberrant TGFβ and SMAD2/3 signaling. It has recently been approved by the American and European medical agencies, based on results from phase 2/3 trials in transfusion dependent MDS patients with ring sideroblasts (RS) or *SF3B1* mutations, refractory or not eligible for ESA [92,93].

#### 5.1.3. Treatment of Thrombocytopenia

Thrombocyte concentrates (TC) are generally transfused prophylactically, if platelets fall 10–20 G/L considering additional factors favoring bleeding (fever or mucositis) or 50 G/L in patients requiring strict anticoagulation [94,95]. While these thresholds are mainly based on experiences from chemotherapy-induced thrombocytopenia, physicians need to be aware of endogenous thrombocyte dysfunctions in MDS favoring bleeding even above such thresholds [96]. Due to short platelet half-life, TC have to be transfused at least weekly or more frequent in conditions of increased consumption (infections) and may be associated with transfusion-related complications (immunization, febrile reactions). Thrombopoietin receptor agonists (TPO-RAs) are established in immunthrombocytopenia (ITP) and represent a possible alternative in MDS patients. Their broader application is hampered by an increase of bone-marrow fibrosis and blast counts observed in clinical trials but, fortunately, without impact on leukemic progression [97,98]. Therefore, TPO-RAs might be safe in lower-risk, but are still not licensed for MDS patients in many countries [47]. Interestingly, recent data also showed an improvement on other cell lines in AA and a combined treatment with immune-suppressive agents might be of value in for hMDS [99].

#### 5.1.4. Treatment of Neutropenia and Infection Prophylaxis

Isolated neutropenia is rare and challenging to treat in lower-risk MDS patients. Most patients with neutropenia have higher-risk disease and may qualify for HMA or more intensive treatment. Evidence is currently insufficient for primary prophylaxis with either G-CSF or anti-infective treatment in patients with severe neutropenia (0.5 G/L) [47]. Secondary prophylaxis with antibiotics, antimycotics or antiviral substances can be made on an individual basis. Even though it has not been systematically investigated, vaccinations against COVID-19, influenza and pneumococci are generally recommended in MDS patients, although the individual immunological response may be very variable [58].

#### 5.1.5. Disease Modifying Treatments in Specific Subsets of Lower-Risk MDS

In transfusion-dependent, lower-risk MDS with del(5q), LEN can provide sustained transfusion independence in two thirds and cytogenetic responses in half of all treated patients, with median duration of transfusion independence of 1–2 years [100]. Patients with mutations in TP53 have shorter response durations and OS [101]. In non-del(5q) MDS patients, the efficacy of LEN seems to be more modest and short lived, but additional benefits have been reported for combined treatment with ESA [91]. Between 5–10% of MDS present with hypoplastic BM, and long-lasting responses have been reported in 16–67% of cases treated with antithymocyte globulin (ATG) combined with cyclosporin A (CyA) with or without TPO-RAs [102].

#### 5.1.6. Hypomethylating Agents

Lower risk MDS patients with predominant neutropenia, multiple cytopenias or who are refractory to first line treatment with growth factors, maturating agents, LEN or other immune-modulating treatments are potential candidates for hypomethylating agents (HMA). HMAs are used in the formal lower-risk setting in the US, but are not licensed in Europe for this indication. Recently, a randomized study compared the safety and efficacy of low-dose HMA (decitabine 20 mg/m^2^ i.v. d1–3 or azacytidine 75 mg/m^2^ i.v. or s.c. d1–3) in lower-risk MDS. The treatment was well tolerated with promising responses ranging between 50–70% in selected patients, but warrants further investigations [103]. Lower-risk MDS patients with symptomatic and refractory cytopenia as well as those with high-risk features have an unmet need for novel treatment options and should be offered clinical trials.

### 5.2. Higher-Risk MDS Patients

#### 5.2.1. Hypomethylating Agents

HMAs comprise the pyrimidine nucleoside analogs 5-azacytidine (AZA) and 5-aza-2′deoxycytidine/decitabine (DEC), the latter only being approved for MDS treatment in the US [104,105]. In a phase 3 trial in higher-risk MDS patients not eligible for allo-HCT, AZA demonstrated significantly higher responses and survival benefit (median OS 24.5 vs. 15.0 months) compared to conventional care regimens with hydroxyurea or low-dose cytarabine (LD-AraC) [104]. Due to delayed HMA treatment response, at least 6 cycles should be administered before considering resistance. HMA remains inferior to more intensive induction chemotherapy followed by allo-HCT, for which, however, only younger and fit patients are eligible [106]. In MDS patients with complex karyotype and lacking of a stem cell donor, HMA should be preferred due to higher CR rates and lower toxicity compared to intensive chemotherapy [107]. Lower HMA response rates have been described in patients with poor performance status (ECOG 2), high transfusion dependency (4 RBCs over 8 weeks), higher number of BM blasts (15%), circulating blasts, higher cytogenetic risk scores and *TP53* mutation. Mutations in epigenetic regulators such as *TET2*, *EZH2* and *DNMT3A* seem to be associated with better responses [108,109,110,111]. Thus far, robust predictive markers for HMA response are lacking. Recently, the FDA granted approval for the combination of oral decitabine and cedazuridine (ASTX727, Inqovi^®^), a cytidine-deaminase inhibitor, as treatment for MDS or CMML, showing equal pharmacokinetic and –dynamic characteristics like the i.v. formulation [112]. As HMA does not significantly modify the clonal disease composition, treatment should be continued as long as tolerated in the absence of signs of progression. Patients refractory to HMA have an unmet need for novel treatments and should be treated within clinical trials.

#### 5.2.2. Induction Chemotherapy

Cytoreductive induction treatment with AML-based chemotherapy before allo-HCT is the mainstay for young and fit higher-risk MDS with ≥10% BM blasts [113]. Alternative induction treatments are fixed liposomal combinations of danorubicine/cytarabine (CPX-351, Vyxeos^®^) [114] or HMA in elderly patients that are deemed to be eligible for allo-HCT, but are at increased risk for toxicity. However, appropriately designed clinical trials to answer the question of the most suitable induction chemotherapy for MDS patients are missing. Good prognostic factors for allo-HCT are younger age, good performance status and favorable cytogenetics [115]. For higher-risk MDS patients with 10% marrow blasts, it remains controversial whether HMA induction is required or if it is better to proceed directly to allo-HCT [113]. For patients without a suitable donor and ≥10% marrow blasts, one course of induction chemotherapy may be recommended followed by HMA maintenance [46]. Patients with poor-risk cytogenetics or *TP53* mutations should be preferentially treated with HMA, as toxicity predominates the limited responses to standard chemotherapy [107]. These patients should be offered induction treatments within clinical trials.

#### 5.2.3. Allogeneic Hematopoietic Stem Cell Transplantation

Allo-HCT remains the only curative treatment option for fit and higher-risk MDS patients up to 75 years of age [113]. As non-relapse mortality depends on comorbidities, the HCT-CI is relevant to estimate non-relapse mortality and the selection of appropriate candidates [116]. Maximal benefit of allo-HCT is associated with transplantation in patients in the higher-risk disease state, whereas lower-risk patients with poor-risk cytogenetic/genetic features, profound cytopenias, and high transfusion burden may also benefit from transplantation [117]. Age, disease status and molecular gene status are the most important predictive factors for OS after allo-HCT, with *TP53*, *RAS* pathway, *ASXL1*, *RUNX1* mutations associated with a higher risk of relapse [118,119,120,121]. Furthermore, NGS-based detection of MRD before conditioning is associated with earlier relapse and might guide the selection of myeloablative or reduced-intensity conditioning [122]. Reduced-intensity conditioning regimens are mainly considered for patients with comorbidities or age 50 years; however, prospective randomized clinical trials have not provided robust evidence for the optimal conditioning regimen [123,124]. Maintenance therapy and MRD-based consolidation therapy should be offered after allo-HCT, whenever possible, in the context of clinical trials [113].

## 6. Ongoing Clinical Trials with Targeting Compounds

The ongoig clinical trials with targeting compounds in lower- and higher-risk MDS are summarized in Table 8.

### 6.1. Lower-Risk MDS

LUSPA is currently investigated as first-line treatment in transfusion dependent, ESA-naïve, lower-risk MDS patients, independent of RS or *SF3B1* mutational status [125]. Roxadustat (FG-4592) is an oral hypoxia-inducible factor prolyl hydroxylase inhibitor (HIF-PHI), which modulates the oxygen-sensing pathway, and increases EPO and erythropoietic output in patients with chronic kidney disease [126]. This compound is currently investigated in a placebo-controlled, phase 3 clinical trial for transfusion-dependent MDS patients in first line [127]. Higher telomerase activity and telomerase reverse transcription (TERT) expression have been identified in mononuclear cells as poor prognostic features in MDS [128,129,130]. This provides the scientific rationale for testing the therapeutic efficacy of the telomerase inhibitor, imetelstat, in MDS. It is a 13-mer oligonucleotide that specifically targets the RNA template of human telomerase, which has been tested in various preclinical studies [131,132,133]. In a recent phase 2 clinical trial, imetelstat increased hemoglobin and reduced transfusion requirements in ESA refractory or ineligible MDS patients. An international phase 3 clinical trial is currently ongoing [133]. Based on the importance of pyroptosis in MDS, an array of inhibitors of S100A8/9, NLPR3 and IL1 have been investigated in pre-clinical models and may enter clinical trials soon. 

### 6.2. Higher-Risk MDS

Spliceosome genes are frequently affected by SLADMs and are mutually exclusive with other spliceosome mutations. Preclinical studies suggest a synthetic lethality of spliceosome inhibitors and a first in class oral spliceosome modulator (H3B-8800) is currently being investigated in higher-risk MDS patients [134,135,136]. Prolonged HMA exposure may improve the efficacy, as these drugs act in the S-phase of the cell cycle. Strategies to achieve this aim include the developments of cytidine deaminase resistant HMAs (guadecitabine) [136,137,138], fixed dose combinations of oral decitabine with the cytidine deaminase inhibitor, cedazuridine [112,139] and oral formulations of AZA (CC-486) [140,141]. The FDA has recently approved oral HMA, CC-486, for maintenance therapy in elderly AML patients, while cedazuridine/decitabine received approval as first line treatment in MDS in the US but not in Europe. These promising agents are currently investigated alone and in combination with different agents in various MDS settings. Combination partners with HMA include immune checkpoint inhibitors (ICI), such as antibodies to CD47 (magrolimab) [142], TIM3 (sabatolimab) [143] and CD70 (cusatuzumab) [144]. Thus far, other ICI targeting PD1, PD-L1 or CTLA4 have shown only limited activity alone and in combination with HMA in higher-risk MDS patients. The BCL2-inhibitor, venetoclax, in combination with HMAs or LD-AraC, has shown substantial activity and has advanced to the standard of care in first-line treatment of elderly patients with AML [145]. This combination has showed promising efficacy also in MDS and is currently further investigated in the first-line and HMA relapsed/refractory settings [146,147,148]. Nevertheless, the management of hematological toxicity remains a major challenge and requires careful monitoring of patients, dose adaptations and supportive treatment with growth-factors, antibiotics, and antimycotics. Neural-precursor-cell-expressed developmentally down-regulated 8 (NEDD8) is an ubiquitin-like protein involved in various DNA repair mechanisms and causes synthetic lethality to cancer cells. In a phase 2 trial in patients with unfit, high-risk MDS/CMML or low-blast count AML, a NEDD8-activating enzyme (NAE) inhibitor (pevonedistat) in combination with HMA showed an improvement of progression-free survival compared to HMA monotherapy [149]. Results from an ongoing phase 3 trial shall be published soon [150]. The discovery of SLADMs has opened a completely new era for risk stratification and patient selection for target therapies. A promising compound is APR-246 (eprenetapopt), a reconfirming agent of mutated *TP53*, which has shown unanticipated responses in *TP53* mutated AML and MDS patients [151,152,153,154]. *IDH1* and *IDH2* mutations occur in ~10% of MDS patients. The corresponding inhibitors (ivosidenib and enasidenib) have shown encouraging results in AML patients and are currently tested in higher-risk MDS setting [155,156,157,158]. Other target therapies for higher-risk MDS, adopted from AML treatment, include the FLT-3 inhibitors (midostaurin, gilteritinib, and quizartinib), which are investigated in phase 2 clinical trials [159,160,161]. Cellular-based immune-therapies are of increasing interest. Chimeric antigen receptor (CAR) T-cell therapy targeting CD123 successfully eliminated MDS stem cells both in vitro and in patient-derived xenografts [162]. Bispecific CD3/CD123 or CD3/CD33 antibodies [154,162,163] as well as personalized adoptive cell therapy, which selects, immunizes and expands T-cells against MDS-specific mutations and targets patient-specific tumor cell neo-antigens, may be promising [164].

## 7. Future Perspectives for Precision Medicine 

### 7.1. Clinical Management Using Guideline-Based Indicators (GBIs)

**Figure 5:**
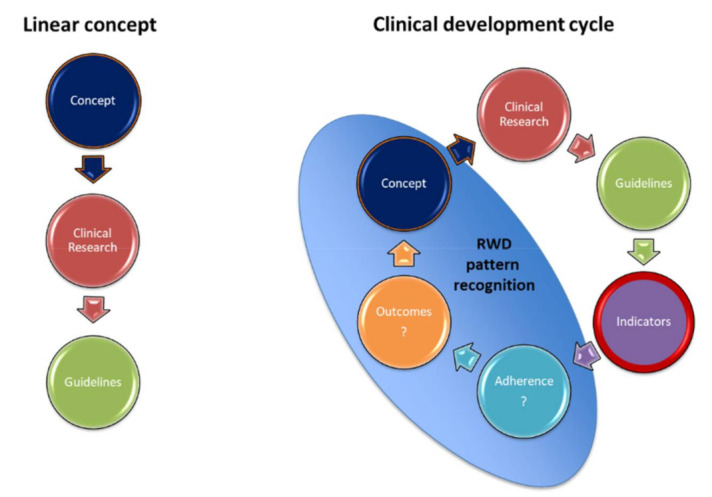
Clinical development cycles. RWD: Real-world data. Currently, clinical development follows generally a liner concept, where clinical research terminates in formulation of guideline and recommendations. Clinical development cycles are iterative, feedback processes, where adherence to guidelines and recommendations and outcomes are continuously assessed using measurable process-based indicators for quality of care.

Diagnosis, prognosis and implications for treatment should be discussed in multidisciplinary boards composed by hemato-oncologists, hematopathologists, radiologist, human geneticists, molecular biologists, clinical pharmacologist, infectiologists, psycho-oncologist, nutritionists and nurses. Moreover, collection of relevant clinical data, outcomes and consensus indicators should be supported by electronic patient charts and may be integrated in clinical quality development cycles [165] (Figure 5). In collaboration with international experts, our study group has developed a first set of 29 relevant guideline-based indicators (GBI) as measurable elements for quality of care for the domains of diagnosis (*n* = 14), treatment (*n* = 8) and provider/infrastructural characteristics (*n* = 7) [47] (Table 9). These GBIs allow the structured and systematic assessment of quality of care in adult MDS patients in different health care environments using real-world data and will eventually help to identify shortcomings for corrective measures.

### 7.2. Diagnosis and Risk-Assessment

New imaging methods based on digital evaluation of PB smears and BM cytomorphology/histopathology slides are increasingly used [166,167,168]. Several study groups aim to develop algorithms for screening, diagnosis, and discrimination from other conditions. Follow-up criteria for MDS patients should be better standardized, including response criteria, assessment of toxicity, functionality and PROs. MRD detection with NGS will be increasingly relevant and may be complemented by single-cell approaches for the discrimination of clonal and normal hematopoiesis, the latter of which may be relevant for prediction of hematopoietic recovery. With the increased armamentarium of therapies, amendable targets such as *TP53*, *IDH1/IDH2*, and *FLT3-ITD/-TKD* should be evaluated in the case of suspected progression, even if these markers were absent at presentation.

### 7.3. Patient Selection for Targeted Therapies

Appropriate patient selection will be critical for implementation of precision medicine in the near future. The fast and reliable generation, interpretation and integration of high-dimensional data will require the implementation of novel structures and technologies coupled to artificial intelligence to support clinical decision-making. Diagnostic procedures and risk-stratification of MDS patients require integration of the most relevant clinical data coupled with morphologic, cytogenetic, molecular genetic as well as omics-approaches, potentially at single cell resolution. As an example, recent studies based on machine learning algorithms suggested combinations of distinct somatic gene mutations or changes in PB values to predict resistance to HMAs [169]. Thus, integrative molecular diagnostics coupled to machine learning approaches may become increasingly important for MDS diagnostics and targeted therapy in the near future [170].

### 7.4. Understanding Clonal Heterogeneity at Single Cell Resolution

The introduction of NGS has facilitated the comprehensive detection of the mutational landscape in MDS. This has also contributed to highlight the complex clonal architecture, explaining the failure of current targeted therapies in achieving clinical cure. Therefore, investigations at single-cell resolution may allow delineating the specific dependencies and vulnerabilities of the mutant and normal stem cells in order to understand individual trajectories of clonal evolution, disease progression or hematopoietic recovery. These approaches could allow the design of more effective differentiation therapies by resolving ineffective hematopoiesis or even targeting minor clones in order to prevent relapse and progression. Prospective national and international registries with associated biobanks should be established to enable comprehensive translational research with real-world data combined to biomarker analyses in large MDS cohorts, as envisaged by our Swiss MDS study group.

## Figures and Tables

**Table 1:** Recurrently mutated leukemia associated driver genes in MDS [11].

Class	Gene	Approx. Frequency (%)
RNA-splicing factors	*SF3B1* *	25–30
	*SRSF2*	10–15
	*U2AF1*	5–10
	*ZRSR2*	5
	*SF3A1*	1–2
	*SF1*	1–2
	*U2AF65*	1–2
	*PRPF40B*	1–2
Epigenetic regulators	*TET2*	20–25
	*DNMT3A*	15
	*ASXL1*	10–15
	*EZH2*	5
	*IDH1*	1–2
	*IDH2*	1–2
Transcription factors	*RUNX1* ^♯^	10–20
	*SETBP1*	1–2
	*ETV6* ^♯^	2
	*CEBPA* ^♯^	1–2
	*GATA2* ^♯^	1–2
Cell-cycle regulators	*TP53*	5–10
	*PTEN*	1
Cohesin complex factors	*STAG1*	1
	*STAG2*	6
	*RAD21*	1
Cell-signaling molecules	*NRAS/KRAS*	5–10
	*NPM1*	1–2
	*JAK2*	1–2
	*FLT3*	2
	*CBL*	1–2

Selection of the most frequent genes affected by recurrent leukemia-associated driver mutations in MDS. Frequencies are only indicative, since they were extracted from studies also including myeloid neoplasms other than MDS. * High association with ring sideroblasts. ^♯^ Somatic as well as constitutional gene mutations.

**Table 2:** Definition of CHIP.

- Absence of relevant cytopenia in the PB
- Evidence of clonality: SLADMs with a VAF of ≥2%
- No evidence of morphologic criteria for any hematologic neoplasm
- PNH, MGUS, and MBL excluded
If relevant cytopenia in PB is present, consider CCUS (minimal diagnostic criteria for MDS are not fulfilled) or MDS (minimal diagnostic criteria are fulfilled). Annual risk of progression to hematologic neoplasm: 0.5–1%

CHIP, clonal hematopoiesis of indeterminate potential; CCUS, clonal cytopenia of undetermined significance; ICUS, idiopathic cytopenia of indeterminate significance; MBL, monoclonal B-lymphocytosis; MDS, myelodysplastic syndrome; MGUS, monoclonal gammopathy of undetermined significance; PB: peripheral blood; PNH, paroxysmal nocturnal hemoglobinuria; SLADMs: somatic leukemia-associated driver mutations; VAF: variant allele frequency. Minimal diagnostic criteria are according to MDS International Working Group (MDS-IWG) [24].

**Table 3:** Landscape of clonal hematopoiesis.

	CHIP [20,21,22]	AA [25,26]	CCUS	MDS [10,11]
Frequency	~2% (40–49 years) ~3% (50–59 years) ~6% (60–69 years) ~10% (70–79 years) ~15% (80–89 years)	19–47% (median age 44 years)	35% of ICUS	70–80% (median age 72 years)
Most common mutations	*DNMT3A*, *TET2*, *ASXL1*, *JAK2*	Younger patients: *PIGA BCOR/BCORL1* Older patients: *DNMT3A*, *ASXL1*	*TET2*, *DNMT3A*, *ASXL1*, *TP53*	*SF3B1*, *TET2*, *ASXL1*, *DNMT3A*, *SRSF2*, *RUNX1*
Prognosis	Increased risk for hematological neoplasm, coronary heart disease, ischemic stroke, diabetes mellitus type 2	Good prognosis: PIGA, BCOR/BCORL1 Poor prognosis: ASXL1, DNMT3A	Increased risk for MDS/AML	Good prognosis: SF3B1 Poor prognosis: TP53, ASXL1, RUNX1, ETV6, EZH2 Neutral: all others
Mean VAF (%)	9%	20% (10% in 40% of patients)	30%	30%
Mean number of mutations per patient	1 (93% of individuals)	1 (64–90% of patients)	1	3 (range 0–12)

AA: Aplastic anemia; CHIP: clonal hematopoiesis of indeterminate potential; CCUS: clonal cytopenia of undetermined significance; ICUS: idiopathic cytopenia of undetermined significance; SLADMs: somatic leukemia-associated driver mutations; VAF: variant allele frequency.

**Table 4:** WHO 2016 classification for MDS.

Subtype ^1^	Number of Dysplastic Lineages	Number of Cytopenic Lineages ^2^	% RS of All Erythroid Cells in BM		% Blasts in PB or BM AR: Auer Rods			Conventional Cytogenetics
			wt*SF3B1*	m*SF3B1*	BM	PB	AR	
MDS-SLD	1	1 or 2	15	5	5	1	-	
MDS-MLD	2 or 3	1–3	15	5	5	1	-	
MDS-RS-SLD	1	1 or 2	≥15	≥5	5	1	-	
MDS-RS-MLD	2 or 3	1–3	≥15	≥5	5	1	-	
MDS del(5q)	1–3	1 or 2	n.a.	n.a.	5	1	-	Isolated del(5q) with or without one additional cytogenetic aberration without del(7) or −7
MDS-EB-1	0–3	1–3	n.a.	n.a.	5–9	2–4	-	
MDS-EB-2	0–3	1–3	n.a.	n.a.	10–19	5–19	+	
MDS-U			15	5	5	1	-	
(a) 1% blasts in PB	1–3	1–3	n.a.	n.a.	5	1 ^3^	-	
(b) SLD with pancytopenia	1	3	n.a.	n.a.	5	1	-	
(c) defining cytogenetic aberration	0	1–3	15 ^4^	n.a.	5	1	-	MDS defining cytogenetic aberration
RCC	1–3	1–3	15	≤5	5	1	-	

^1^ Without previous cytotoxic treatment or germline predisposition for myeloid neoplasms; ^2^ Cytopenias: hemoglobin 100 g/L, thrombocytes 100 G/L, neutrophils 1.8 G/L, monocytes 1 G/L; ^3^ One percent blasts in PB must be confirmed with a second measurement; ^4^ ≤15 RS corresponds to MDS-RS-SLD. CAVE: If ≥50% are erythroid precursors and ≥20% blast cells of non-erythroid-lineage but 20% of all cells, this corresponds to MDS (MDS-SLD/MLD or EB) and not to AML M6 erythroid/myeloidAR, Auer rods; BM: bone marrow; del(5q), loss of part of the long arm of chromosome 5; EB, excess of blasts; MDS, myelodysplastic syndromes; MDS-U, myelodysplastic syndromes unclassified; MLD, multi-lineage dysplasia; PB, peripheral blood; RCC, refractory cytopenia of the childhood; RS, ring sideroblasts; SLD, single lineage dysplasia; wt/m*SF3B1*, wild-type or mutated *SF3B1.*

**Table 5:** Morphological characteristics of dysplasia [49].

Peripheral Blood
- Erythrocytes: anisocytosis - Neutrophils: Pseudo-Pelger--Huët anomaly, cytoplasmic hypogranularity
- Thrombocytes: anisocytosis, giant platelets
Bone marrow
- Dyserythropoiesis: nuclear budding, internuclear-bridging, karyorrhexis, multinuclearity, megaloblastoid changes, ring sideroblasts, vacuolization, periodic acid-Schiff (PAS) positivity
- Dysgranulopoiesis: small or unusually large size, nuclear hypo- or hypersegmentation, decreased granules/agranularity, Pseudo-Chédiak--Higashi granules, Döhle bodies, Auer rods - Dysmegakaryopoiesis: micromegakaryocytes, nuclear hypolobation, multinucleation

**Table 6:** Minimal diagnostic criteria for the diagnosis of MDS ([24]).

Criteria	Diagnostic Test
1. Mandatory criteria (both have to be fulfilled)	
Persistent cytopenia(s) for more than 4 months *	PB counts and morphological assessment
Exclusion of other disease(s) that may cause cytopenia/dysplasia	BM aspirate and biopsy, cytogenetics, flow cytometry, molecular genetics, other relevant investigations **
2. MDS-defining criteria (at least one has to be fulfilled)	
Morphological criteria of dysplasia 10% in at least one cell lineages investigated in the BM	PB, BM aspirate and biopsy
Blasts 5–19% in BM or 2–19% in PB	PB, BM aspirate and biopsy
Ring sideroblasts ≥15% or ≥5% with SF3B1 mutation	Iron staining, sequencing
MDS-defining cytogenetic alterations ***	Conventional metaphase cytogenetics, interphase fluorescence in situ hybridization, array comparative genomic hybridization
3. Co-criteria (for patients with 1. but not 2., two have to be fulfilled)	
Abnormal findings in histologic and/or immunohistochemical studies of supporting the diagnosis of MDS ****	BM biopsy sections with immunohistochemistry
Abnormal immunophenotype of BM cells with aberrant immunophenotype indicative for a monoclonal population	Flow cytometry
Clonality of myeloid cells revealing MDS-related mutations	Molecular genetics, next generation sequencing

If no major criterion is fulfilled, but the patient is likely to suffer from a clonal myeloid disease, co-criteria should be applied and may help in reaching the conclusion that the patient has a myeloid neoplasm resembling MDS or will develop MDS. In this diagnostic setting, repeated bone marrow investigations during follow-up may be required to arrive at a final diagnosis of MDS. BM: bone marrow; PB: peripheral blood. * Cytopenia defined by local institutional reference values. ** Investigations depend on individual criteria and should include serum electrophoresis with immunofixation, erythropoietin and tryptase. *** Cytogenetic alterations indicative of MDS, as defined by WHO. **** i.e., clusters of abnormally localized immature precursors (ALIP); clusters of CD34+ blast cells; dysplastic micromegakaryocytes detected by immunohistochemistry (≥10% dysplastic megakaryocytes).

**Table 7:** Integrated cyto-histologic/genetic score for distinction of non-malignant bone-marrow failure syndromes and hypoplastic MDS.

Requisite Criteria	Score
BM blasts AND/OR CD34+ cells ≥5%	2
BM blasts AND/OR CD34+ cells 2–4%	1
Fibrosis grade 2–3	1
Dysmegakaryopoiesis	1
Co-criteria	
Ring sideroblasts ≥15%	2
Ring sideroblasts 5–14% *	1
Severe dysgranulopoiesis	1
Karyotype (co-criterion)	
Presumptive cytogenetic abnormality *	2
Somatic mutation (co-criterion)	
Specific high-risk mutation pattern **	1

* According to WHO 2016 criteria (Table 4) [48]. ** According to Malcovati et al. [53]; BM: bone marrow; hg-score: cyto-histologic/genetic score; ROC analysis confirmed that a cutoff hg-score of 2 is associated with the highest percentage of correctly classified hMDS cases (AUC 0.89, *p* 0.001).

**Table 8:** Overview on novel targeted and immunotherapeutic options for patients with MDS.

Compound	Study Design	Efficacy *	Safety *	NCT
Erythropoiesis maturating agents				
TGFβi luspatercept	Phase 3 (ongoing), open-label, randomized study: efficacy and safety of luspatercept (ACE-536) versus epoetin alpha for the treatment of anemia due to IPSS-R very low, low or intermediate risk according to IPSS-R MDS in ESA naïve subjects requiring red blood transfusions (COMMANDS)	ongoing 38% vs. 13% TI for 8 weeks or longer	AE: - similar grade 3/4: 42% vs. 45%, 5% doses reduction - frequent: fatigue, diarrhea, asthenia, nausea, - dizziness, back pain	NCT03682536 [125]
Hypoxia-inducible factor prolyl hydroxylase inhibitor (HIF-PHi)				
roxadustat (FG-4592)	Phase 3, randomized double-blind placebo-controlled study investigating the efficacy and safety of roxadustat (FG-4592) for treatment of anemia in patients with lower risk MDS with low RBC transfusion burden	ongoing	ongoing	NCT03263091 [127]
Telomerase inhibitor				
imetelstat	Phase 2/3 (phase 3 part ongoing), double-blind, randomized study to evaluate imetelstat (GRN163L) versus placebo in transfusion-dependent subjects with IPSS low or intermediate-1 risk MDS that is relapsed/refractory to ESA treatment (IMERGE)	TI in 8- and 24-week: 37%, respectively 23%, median duration of 65 weeks phase 3 ongoing	AE: - cytopenias, typically reversible within 4 weeks	NCT02598661 [133]
Spliceosome modulators				
H3B-8800	Phase 1 (ongoing), open-label trial to evaluate the safety, pharmacokinetics and pharmacodynamics of splicing modulator H3B-8800 for subjects with MDS, AML and CMML	decreased RBC or TC requirement in 14%	AE: - diarrhea: 75% - nausea: 37% - fatigue: 28% - vomiting: 27%	NCT02841540 [135,136]
DNA-methylation				
guadecitabine (SGI-110)	Phase 1/2, dose escalation, dose escalation, randomized study of two regimens of SGI-110, in subjects with intermediate or high-risk MDS or AML	ORR 40% with 60 mg/m^2^ d1-d5 q28d ORR 55% with 90 mg/m^2^ d1-d5 q28d	AE: - febrile neutropenia: 11% - pneumonia: 7% - anemia: 3% - thrombo- cytopenia: 3% - 2 deaths (pneumonia, septic shock)	NCT01261312 [112]
guadecitabine (SGI-110)	Phase 3, randomized, open-label study of SGI-110 versus treatment choice in adults with MDS or CMML previously treated with hypomethylating agents	primary endpoint (OS): no statistically significant improvement secondary endpoints: analysis ongoing	comparable to previous studies	NCT02907359 [138]
oral AZA/ cedazuridine (CDZ) (ASTX030)	Phase1 (ongoing), multi-phase, dose-escalation followed by an open-label, randomized, crossover study of oral ASTX030 versus subcutaneous azacytidine in subjects with MDS, CMML or AML	parenteral and oral AZA + CDZ similar pharmacokinetic profiles and efficacy against human AML cells	n.a.	NCT04256317 [139]
oral DEC/CDZ (ASTX727)	Phase1/2 pharmacokinetic guided dose-escalation and dose-confirmation study of ASTX727 (oral cytidine deaminase inhibitor E7727 with oral decitabine) in subjects with MDS	no difference in pharmakokinetics, pharmacodynamics and efficacy between p.o. and i.v. formulations	similar between p.o. und i.v.	NCT02103478 [112]
Immune checkpoint inhibitors				
CD47-Ab magrolimab	Phase 1b Trial of magrolimab monotherapy versus in combination with azacytidine in patients with hematological malignancies	TI: 58% (MDS), 64% (AML) objective response (CR, marrow CR, HI): 91% (MDS)	AE: - anemia: 38% - neutropenia: 19%, - thrombocytopenia: 18% - infusion reaction 16%	NCT03248479 [142]
TIM3-Ab sabatolimab (MBG453)	Phase 1b, multi-arm, open-label study of PDR001 and/or MBG453 in combination with decitabine in patients with AML or high risk MDS	AML: ORR 41.2% MDS: ORR 62.9%	AE: Grad 3/4 (AML/MDS): - thrombocytopenia (45.8%, 51.2%) - neutropenia (50%, 46.1%) - febrile neutropenia (29.2%, 41%) - anemia (27.1%, 28.2%) - pneumonia (10.4%, 5.1%)	NCT03066648 [143]
Proapoptotic agents				
BCL-2i venetoclax + AZA	Phase 1b/2 (ongoing), dose escalation study evaluating the safety and pharmacokinetics of venetoclax in combination with azacytidine in subjects with treatment-naïve higher-risk MDS	18 months: OS 74%, HI 50%	AE, grade 3/4: - neutropenia (68%) - febrile neutropenia (46%) - thrombocytopenia (39%) - anemia (19%)	NCT02942290 [147]
BCL-2iv enetoclax + AZA	Phase 1b study (ongoing) evaluating the safety and pharmacokinetics of venetoclax as a single-agent and in combination with azacytidine in subjects with relapsed/refractory MDS	median FU 4.7 months: - monotherapy: ORR 7%, SD 75%, - PFS 3.4 months, OS 6 months 57% - combination: ORR 50%, SD 31%, - PFS/OS not reached	Grade 3/4: - neutropenia (41%) - febrile neutropenia (17%) - pneumonia (13%) - thrombocytopenia (30%) - anemia (15%)	NCT02966782 [148]
NEDD8i pevonedistat	Phase 2, randomized, controlled, open-label, study of the efficacy and safety of pevonedistat plus azacytidine versus single-agent azacytidine in patients with higher-risk MDS, CMML and low-blast AML	Combination vs. single-arm - OS 23.9 versus 19.1 months - ORR 79.3% vs. 56.7% - CR^1^ 51.7% versus 26.7%	Grade 3/4 adverse events similar (69% vs. 63% in single-arm) - neutropenia 33% vs. 27% - febrile neutropenia 26% vs. 29% - thrombocytopenia 19% vs. 23% - anemia 19% vs. 27%	NCT02610777 [149]
NEDD8i pevonedistat	Phase 3, randomized, controlled, open-label, study of pevonedistat plus azacytidine versus single-agent azacytidine as first-line treatment for patients with Higher-Risk MDS, CMML or low-blast AML (PANTHER)	ongoing	ongoing	NCT03268954 [150]
*TP53* reconforming agents				
eprenetapopt (APR-246)	Phase 1b/2 (ongoing) study to evaluate the safety and efficacy of APR-246 in combination with azacytidine for the treatment of TP53 mutant myeloid neoplasms	ongoing	Grade 3/4 (phase 1b): - neutropenia 42% - thrombocytopenia 50%	NCT03588078 [154]
Epigenetic inhibitors				
IDH1i ivosidenib (AG-120)	Phase 1, open-label, dose-escalation and expansion, safety, pharmacokinetic, pharmacodynamic, and clinical activity study of orally administered AG-120 in subjects with advanced hematologic malignancies with an *IDH1* mutation	CR^2^ plus CRh^2^: 42.4% CR^2^ 30.3% FU 23.5 months: median OS 12.6 months TI in 9 of 21 TD (42.9%) *IDH1* mutation clearance in 9/14 patients	AE: - All grades: diarrhea (53%), fatigue (47%), nausea - (38%), decreased appetite (35%), DS (18%) - Grade ≥ 3 (9%): DS, QTc-prolongation, febrile - neutropenia, diarrhea (did not require treatment discontinuation)	NCT02074839 [157]
IDH2i enasidenib (AG-221)	Phase 1/2 (ongoing), open-label, dose-escalation and expansion, safety, pharmacokinetic, pharmacodynamic and clinical activity study of orally administered AG-221 in subjects with advanced hematologic malignancies with an *IDH2* mutation	phase 1: ORR 53% median duration 9.2 months OS 16.9 months phase 2 ongoing	Grade 3/4 (phase 1): - indirect hyperbilirubinemia (35%), pneumonia (29%) - thrombocytopenia (24%), tumor lysis - syndrome (18%), sepsis (12%), atrial flutter (12%), - cerebral hemorrhage (12%), mental status change (12%) - no treatment-related deaths	NCT01915498 [158]
*RAS*-pathways inhibitors				
FLT3i quizartinib (AC220)	Phase 1/2 study of the combination of quizartinib (AC220) with 5-azacytidine or low-dose cytarabine for the treatment of patients with AML and MDS	quizartinib/AZA-arm vs quizartinib/LD-AraC-arm: - previously untreated patients, CRc: 87% vs. 74% OS: 19.2 vs. 8.5 months - previously treated patients CRc: 64% vs. 29% - OS: 12.8 vs. 4 months	AE grade 3/4, quizartinib/AZA-arm vs. quizartinib/LD-AraC-arm: - neutropenia: 5% vs. 42% - febrile neutropenia: 40% vs. 36% - anemia: 0% vs. 15% - thrombocytopenia: 13% vs. 30% - pneumonia: 28% vs. 48% - hypokalemia: 33% vs. 9% - Hyperbilirubinemia: 15% vs. 3%	NCT01892371 [161]
Bispecific antibodies				
CD3/CD123 Flotetuzumab (MGD006)	Phase 1/2, first in human, dose escalation study of MGD006, a CD123 × CD3 DART^®^ bi-specific antibody based molecule, in patients with relapsed or refractory AML or intermediate-2/high risk MDS	CR/CRh: 26.7% median OS 10.2 months ORR (CR/CRh/CRi): 30%	most frequent AE: - infusion-related reactions (IRRs) and cytokine release syndrome (CRS), largely grade 1–2	NCT02152956 [163]
CD3/CD33 (AMG 330)	A Phase 1 first-in-human study evaluating the safety, tolerability, pharmacokinetics, pharmacodynamics and efficacy of AMG 330 administered as continuous intravenous infusion in subjects with myeloid malignancies	ongoing	ongoing	NCT02520427 [154]

* Efficacy and safety data are extracted from published studies in AML/MDS according to the references. CR^1^: complete remission/HI: hematological improvement per International Working Group (IWG) 2006 criteria for myelodysplastic syndromes (MDS), CR^2^: complete remission according to IWG 2003 response criteria for AML, CRc: composite response (CR^2^ + CRi + CRp + CRh), CRi: CR^2^ with incomplete hematologic recovery, CRh: CR^2^ with partial hematological recovery (bone marrow myeloblasts of 5% combined with both absolute neutrophil count 500/μL and platelet count 50 × 10^9^/L, CRp: CR^2^ without platelet recovery, DS: differentiation syndrome, FU: follow-up, ORR: overall response rate, OS: overall survival, PFS: progression free survival, RBC: red blood cells, SD: stable disease, TI: transfusion independency, TD: transfusion dependent.

**Table 9:** Guideline-based indicators for adult MDS patients.

Domain 1 Diagnosis (*n* = 14)	Domain 2 Therapy (*n* = 8)	Domain 3 Provider/Infrastructural Characteristics (*n* = 7)
Diagnostic work-up: - Cytogenetic analysis - BM cytology/histology - PB assessment - WHO 2016 classification - Iron staining/monitoring - Serum EPO in symptomatic anemia - Molecular diagnostics/NGS - TP53 in MDS del(5q) Risk stratification: - Disease-based risk stratification - (IPSS/IPSS-R) - Patient-based risk stratification - (Karnovsky, ECOG, HCT-CI, MDS-CI) Follow-up/outcomes: - Response assessment - (IWG criteria including LFS, OS) - Patient-reported outcomes (PROs)	Supportive care: - Transfusions of RBCs - Transfusions of TCs Lower-risk: - Erythropoietin stimulating agents (ESA) - Lenalidomide in MDS del(5q) Higher-risk (unfit patients): - Hypomethylating agents (HMAs) Higher-risk (fit patients): - Induction before allo-HCT - (blasts ≥10%) - Up-front allo-HCT (blasts 5–10%) - Allo-HCT	Personnel: - Multidisciplinary care team Organization: - Safe handling of cytotoxic drugs - Interdisciplinary diagnostic review - Interdisciplinary treatment board - Teaching and continuing education - Emergency services Cooperation: - Access to clinical trials

Allo-HCT: allogeneic hematopoietic stem cell transplantation; BM: bone marrow; EPO: erythropoietin; ESA: erythropoietin stimulating agents; HCT-CI: hematopoietic cell transplantation-specific comorbidity index; IPSS/IPSS-R: international prognostic scoring system and revised IPSS; IWG: international working group; LFS: leukemia free survival; OS: overall survival; MDS-CI: MDS-specific comorbidity index; NGS: next generation sequencing; PB: peripheral blood; PROs: patient-reported outcomes; RBCs: red blood cell concentrates; TC: thrombocyte concentrates.

## Abbreviations

ACMG	American College of Medical Genetics
Allo-HCT	Allogeneic hematopoietic stem cell transplantation
ARCH	Age-related clonal hematopoiesis
ATG	Antithymocyte globulin
AML	Acute myeloid leukemia
BM	Bone marrow
CCUS	Clonal cytopenia of undetermined significance
CGH	Comparative Genomic Hybridization
CHIP	Clonal hematopoiesis of indeterminate potential
CsA	Cyclosporine A
EMA	Erythroid Maturation Agents
EPO	Erythropoietin
ESA	Erythropoietin Stimulating Agents
FISH	Fluorescent in situ hybridization
GBIs	Guideline-based indicators
G-CSF	Granulocyte Colony-Stimulating Factor
G/Rs	Guidelines and recommendations
HCT-CI	Hematopoietic Cell Transplantation Comorbidity Index
HI	Hematological improvement
HMA	Hypomethylating agents
HSPC	Hematopoietic stem and progenitor cells
HU	Hydroxyurea
ICI	Immune checkpoint inhibitor
ICUS	Idiopathic cytopenia of undetermined significance
IDUS	Idiopathic dysplasia of undetermined significance
IP	Immunophenotyping
IPSS	International Prognostic Scoring System
IPSS-R	Revised International Prognostic Scoring System
IST	Immunosuppressive treatment
LD-AraC	Low-dose cytarabine
LEN	Lenalidomid
LUSPA	Luspatercept
MDS	Myelodysplastic syndromes
MRD	Measurable residual disease
NGS	Next generation sequencing
OS	Overall survival
PB	Peripheral blood
RBC	Red blood cell concentrates
sAML	Secondary AML
SLADMs	Somatic leukemia-associated driver mutations
TC	Thrombocyte concentrates
TLR	Toll-like receptor
TPO-RA	Thrombopoietin receptor agonists
QoL	Quality of life
VAF	Variant allele frequency
WHO	World Health Organization
WPSS	WHO Prognostic Scoring System
